# Traditional Medicines in the World: Where to Go Next?

**DOI:** 10.1155/2014/739895

**Published:** 2014-12-29

**Authors:** Si-Yuan Pan, Gerhard Litscher, Kelvin Chan, Zhi-Ling Yu, Hou-Qi Chen, Kam-Ming Ko

**Affiliations:** ^1^Department of Pharmacology, School of Chinese Materia Medica, Beijing University of Chinese Medicine, Beijing 100102, China; ^2^Research Unit for Complementary and Integrative Laser Medicine, Research Unit of Biomedical Engineering in Anesthesia and Intensive Care Medicine, and TCM Research Center Graz, Medical University of Graz, 8036 Graz, Austria; ^3^Faculty of Pharmacy, University of Sydney, Sydney, NSW 2006, Australia; ^4^School of Chinese Medicine, Hong Kong Baptist University, Kowloon Tong, Kowloon, Hong Kong; ^5^American Academy of Natural Medicine, Costa Mesa, CA 92627, USA; ^6^Division of Life Science, Hong Kong University of Science & Technology, Clear Water Bay, Hong Kong

According to the WHO, 65–80% of the world's healthcare practice involves the use of traditional medicine (TM), commonly referred to as complementary and alternative medicine (CAM), in some way. Today, TM has become an indispensable part of our health management. It has been well known that TM covers a wide array of therapies and practices which vary from culture to culture and country to country. It is more important that TM differs from conventional medicine (CM) in both theory and practice. However, the research and development of almost all TM systems mostly follow the track that had been laid down by CM nowadays. It is clearly not appropriate for the future development of TM. Therefore, a well-structured strategy for research, practice, and development is instrumental to optimize the utilization of TM which reflects its superiority over CM in some ways. Now it is time for us to start thinking about “where is TM heading?” and “how should TM reach its destination?”

This special issue focuses on TM development and practice strategy in the theory and experiment.

N. T. Y. Leung et al. contributed a paper entitled “*Potential therapeutic effects of meditation for treating affective dysregulation*.” Affective dysregulation is at the root of many psychopathologies. The root of these disorders appears to be an attenuated, top-down cognitive control from the prefrontal cortices over the maladaptive subcortical emotional processing. The mainstream treatment approaches are antidepressant medication and cognitive behavioral therapy. Because of its emphasis on present-moment awareness and acceptance, a growing body of research has explored the potential clinical applications of meditation for disorders stemming from affective dysregulation. Current evidence reveals that meditation may prevent and intervene in mood and other affective disorders. Increasing research has suggested that the cultivation of awareness and acceptance along with a nonjudgmental attitude via meditation promotes adaptive affective regulation. This review examined the concepts of affective regulation and meditation and discussed behavioral and neural evidence of the potential clinical application of meditation. Lately, there has been a growing trend toward incorporating the* mindfulness* component into existing psychotherapeutic treatment. Promising results have been observed thus far. Future studies may consider exploring the possibility of integrating the element of* compassion* into current psychotherapeutic approaches.

In their paper entitled “*Acupuncture and depth: future direction for acupuncture research*,” Y. L. Goh et al. concluded that the plentiful variables that exist in acupuncture research are the reason for the lack of scrutiny on the depth of acupuncture. The authors stated that, as research progresses, attention needs to be given to the amount of tissue invaded and types of tissues excited. Along with the advancements in imaging, laser technologies, and so forth, research on acupuncture depth could also progress. They suggest that future acupuncture research, especially RCTs, should take into consideration the depth of insertion. In addition, the use of bibliometric method is crucial for future development of traditional medicine research trends too.

The paper entitled “*Antidiabetic and antioxidative effect of Jiang Tang Xiao Ke granule in high-fat diet and low-dose streptozotocin induced diabetic rats*” by D.-D. Zhao et al. deals with diabetes mellitus (DM), a kind of metabolic disease, which has been increasing over the last four decades in the world. The purpose of their study was to investigate the effect of Jiang Tang Xiao Ke (JTXK) granule, a naturally occurring ingredient from Chinese herbal medicines, on serum glucose, lipids, and oxidative stress in DM rats induced by high-fat diet and streptozotocin. In conclusion, they found that JTXK granule, at 9 g/kg (based on crude herbal material), treatment for 4 weeks reduced serum glucose via increasing insulin sensitivity and protection of pancreas islets in DM rats. In addition, the JTXK granule decreased serum total cholesterol, triglyceride, and low density lipoprotein (LDL) levels but increased high density lipoprotein (HDL) levels, compared with the drug-untreated DM rats. At the same time, JTXK granule showed improved antioxidant activity, which was manifested by decreased malondialdehyde and nitric oxide levels and with elevation in superoxide dismutase levels in DM rats. Islet morphology showed marked improvement in DM rats treated with JTXK granule. These findings suggested that JTXK granule may be an effective and safe alternative treatment for type 2 DM.

S. Telles et al. wrote the paper entitled “*Research on traditional medicine: what has been done, the difficulties, and possible solutions*.” They stated that traditional medicine (TM) is being used more frequently all over the world. However, most often, these are choices made by the patient. Integrating TM into mainstream healthcare would require research to understand the efficacy, safety, and mechanism of action of TM systems. This paper describes research done on TM and difficulties encountered in researching TM, especially when an attempt is made to conform to the model for conventional medicine. The last part of the report provides suggestions to make research on TM more acceptable and useful, with the ultimate goal of integrating TM into mainstream healthcare with sufficient knowledge about the efficacy, safety, and mechanism of action of TM systems.

The contribution by H. Zhang et al. is entitled “*A disturbance rejection framework for the study of traditional Chinese medicine.*” In this paper, traditional Chinese medicine (TCM) is explained in the language of engineering cybernetics (EC), an engineering science with the tradition of rigor and a long history of practice. The inherent connection is articulated between EC, as a science of interrelations, and the Chinese conception of Wuxing. The combined cybernetic model of Wuxing seems to have significant explaining power for the TCM and could potentially facilitate better communications of the insights of the TCM to the West. EC and TCM also share commitment and goal to attain and maintain a quality that is described as balance, equilibrium, or homeostasis; correspondingly, there is a great similarity in how such quality is maintained: by what is called “disturbance rejection” in EC. In disturbance rejection, a great metaphor is found to show how TCM is practiced, using a case study as illustration. The results from this six-year study seem to support this line of investigation and to provide a clear exposition of both the principles of the TCM and how they are practiced with herbal medicine. It is the authors' hope that what started in this paper could help future researchers to build the bridge from the general system qualities and behaviors to the properties of the system components.

X.-Y. Wang et al. dealt with “*Supplementation with the extract of Schisandrae Fructus pulp, seed, or their combination influences the metabolism of lipids and glucose in mice fed with normal and hypercholesterolemic diet*.” Schisandrae Fructus (SF), which possesses five tastes, can produce a wide spectrum of biological activities in the body. In this paper, the authors investigated the effects of the ethanolic extract of SF pulp, seed, or their combination (namely, EtSF-P, EtSF-S, or EtSF-P/S, resp., collectively called EtSF) on the metabolism of lipids and glucose in normal diet- (ND-) and hypercholesterolemic diet- (HCLD-) fed mice. In conclusion, results showed that the supplementation with EtSF produced a significant influence on lipid/glucose metabolism in ND- and HCLD-fed mice, especially in hypercholesterolemic (HCL) mice. Dietary supplementation with EtSF-P or EtSF-S/P elevated serum lipid levels, except for that of serum triglyceride levels which was lowered, in HCL mice. Dietary supplementation with EtSF-S, EtSF-P, or EtSF-S/P reduced hepatic lipid and glucose concentrations in both normal and HCL mice. EtSF-S/P, but not EtSF-S or EtSF-P, supplementation increased fecal cholesterol excretion in HCLD-fed mice. EtSF-P and EtSF-S/P attenuated the HCLD-induced hepatotoxicity. Supplementation with fenofibrate decreased serum and hepatic lipid and glucose levels and increased serum ALT activity and liver weight in mice fed with ND and/or HCLD. The ensemble of results indicates a differential effect between SF seed and pulp on lipid and glucose metabolism, particularly in HCL mice. Thus, the authors concluded that supplementation with EtSF might ameliorate the lipid accumulation in liver cells and thus protect against liver injury in HCL mice.

The paper entitled “*Historical perspective of traditional indigenous medical practices: the current renaissance and conservation of herbal resources*” by S.-Y. Pan et al. takes a thorough look at Chinese, Indian, and Arabic herbal medicine over the centuries. It was found that, based on cultures and geographical regions, various kinds of herbal remedies have evolved. Herbal medicines are therefore an integral part of culture and geographical environment, and various kinds of herbal medicines have their own unique way of understanding and treating a disease. However, the globalization of trade and market has brought about an integration of different kinds of herbal medicines over the world. At present, herbal medications or related products in the global market are derived from Chinese herbs, Indian herbs, Arabic herbs, and Western herbs. Herbal remedies may be also classified into three categories, namely, modern herbs, theoretical herbs, and empirical herbs, in accordance with their nature/characteristics and the nature of current usage. In general, most herbal remedies/formulae are considered to be safe and are well tolerated because they have been successfully used for thousands of years as foods to promote health and as medicines to treat diseases. To date, herbal products are widely available to consumers and have become increasingly popular throughout the world. For the authors, there is no doubt that herbal products will continue to play a crucial role in the healthcare system of human societies, not to mention that secondary metabolites of plants are economically important as drugs, fragrances, cosmetics, food additives, and pesticides.

I. O. Lawal et al. obtained “*Phytotherapeutic information on plants used for the treatment of tuberculosis in Eastern Cape province, South Africa*,” because the current rate of deforestation in Africa constitutes a serious danger to the future of medicinal plants on this continent. Conservation of these medicinal plants in the field and the scientific documentation of our knowledge about them are therefore crucial. An ethnobotanical survey of plants used for the treatment of tuberculosis (TB) was carried out in selected areas of the Eastern Cape, South Africa. The documented medicinal plants used by the Xhosa herbalist reflect a rich ethnomedicinal knowledge in the municipality. These results strengthen the firm belief that traditional medicines are readily accessible and still play an important role in meeting the basic healthcare of many people in African communities. Phytomedicinal information on the treatment of TB in this region is well established. Thirty plants belonging to 21 families were mentioned to be used for the treatment of TB and associated diseases. Other diseases treated using these plants were respiratory infections. The commonly mentioned species are* Clausena anisata*,* Haemanthus albiflos, Artemisia afra, Carpobrotus edulis, Ptaeroxylon obliquum, *and* Tulbaghia violacea. *The most frequently mentioned species was* Clausena anisata, *known locally as* Iperepes*. Many studies have revealed some bioactive chemical compositions in these plants which probably justify their pharmacological properties. The following five species were recorded for the first time for the management of TB in Eastern Cape province, South Africa:* Clausena anisata, Haemanthus albiflos, Araujia sericifera, Scabiosa albanensis, and Silene undulata. *This study has contributed to the scientific documentation of medicinal plants used for the treatment of TB. This is necessary in the rural communities to avert the erosion of traditional medicine knowledge. The larger percentage of the traditional healers are old people; therefore, this legacy needs to be conserved. Finally, the authors announced that further studies are in progress on the antituberculosis assay to validate ethnopharmacology relevance of the most mentioned plants in the study area.

The paper by X.-Q. Wang et al. is entitled “*New perspectives on specific immune-depletion technique using monoclonal antibodies against small active molecules in herbs*.” One of the main focuses in Chinese medicine research is the identification of efficacious components in Chinese herbal medicine (CHM). According to the authors, current methods to track and separate active components are not adequate to meet the needs of revealing effects and identify substances and pharmacological mechanisms, which directly restrict the modernization and globalization of CHM. Thus, they introduced a new methodology to deplete a single active component via immunoassay. The specific active component in a CHM mixture can then be identified and studied through comparative analyses of the pharmacological effects before and after immune-depletion. These tools allow researchers to have a clear understanding on how to conduct a more in-depth study in CHM research.* Radix puerariae *was used as an example for demonstrating monoclonal antibody technology using mAb deletion. By comparing the pharmacological properties and pharmacodynamics of* Pueraria* extract before and after undergoing puerarin immunodepletion, the main contribution of puerarin to the overall medicinal property of* Pueraria* can then be easily evaluated, in vivo and in vitro, static and dynamic. As a result, puerarin can then be better utilized in QC, compatibility studies, and pharmacokinetic studies of* Pueraria* roots, which expands the usage of* Pueraria* roots in clinical medicine in the future. And also as a result, the experimental foundation for any future CHM studies using target specific deletion techniques was established. However, the methodology introduced in this paper still has its limitations. Taking everything into account, Wang et al. conclude that the new research strategy still holds many advantages and has proven to provide more effective and convenient ways of pursuing CHM research and help the modernization of Chinese medicine.

The paper entitled “*Ethyl acetate extract of Artemisia anomala S. Moore displays potent anti-inflammatory effect*” was authored by X. Tan et al. They state that* Artemisia anomala *S. Moore has been widely used in China to treat inflammatory diseases for hundreds of years. However, mechanisms associated with its anti-inflammatory effect are not clear. In this study, they prepared ethyl acetate, petroleum ether,* n*-BuOH, and aqueous extracts from ethanol extract of* Artemisia anomala *S. Moore. Comparing anti-inflammatory effects of these extracts, they found that ethyl acetate extract of this herb (EAFA) exhibited the strongest inhibitory effect on nitric oxide (NO) production. EAFA suppressed the production of NO in a time- and dose-dependent manner without eliciting cytotoxicity. To understand the molecular mechanism underlying EAFA's anti-inflammatory effect, the authors showed that EAFA increased total cellular antioxidant capacity while reducing the amount of inducible nitric oxide synthase (iNOS) in stimulated RAW264.7 cells. EAFA also suppressed the expression of IL-1*β* and IL-6, whereas elevating the level of heme oxygenase-1(HO-1). These EAFA-induced events were apparently associated with NF-*κ*B and MAPK signaling pathways because the DNA binding activity of p50/p65 was impaired and the activities of both ERK and JNK were decreased in EAFA-treated cells comparing to untreated cells. Their findings suggest that EAFA exerts its anti-inflammatory effect by inhibiting the expression of iNOS. In conclusion, this study indicates that EAFA can potently suppress inflammatory responses.

In their paper entitled “*New insights into the chemical and biochemical basis of the* “*Yang-invigorating” action of Chinese Yang-tonic herbs*,” J. Chen et al. propose a biochemical mechanism of “Yang-invigorating” action produced by Yang-tonic herbs. Triterpenes or phytosterols, the active components in Yang-tonic herbs with similar chemical structure, can stimulate mitochondrial electron transport through the increase in the fluidity of the inner mitochondrial membrane, with a resultant increase in ATP generation capacity. The stimulation of electron transport is accompanied by an increase in ROS production, which triggers a retrograde response to upregulate cellular/mitochondrial antioxidant defense mechanisms through the Nrf2 pathway. In addition, they found that mitochondrial ROS can also stimulate the activity of uncoupling protein and thereby lower the membrane potential through the dissipation of proton gradient. With a recurring “Yang-invigorating” action produced by the active components of Yang-tonic herbs, an intermittent stimulation of mitochondrial ROS production at low levels results in the prolonged activation of mitochondrial uncoupling and the upregulation of antioxidant defense components characteristic of mitohormesis, which is beneficial in retarding the decline in body function and delaying the onset of age-related diseases.

